# 958. A Required Infectious Diseases Rotation Improves Antimicrobial Stewardship Knowledge for Internal Medicine Interns

**DOI:** 10.1093/ofid/ofab466.1153

**Published:** 2021-12-04

**Authors:** Lee S Gottesdiener, Kate Stoeckle, Angela Loo, Shawn Mazur, Kirana Gudi, Kristen Marks, Kristen Marks, Matthew Simon

**Affiliations:** 1 New York-Presbyterian Hospital, New York, New York; 2 Weill Cornell Medicine, New York, New York

## Abstract

**Background:**

Exposure to Infectious Diseases (ID) education is highly variable in post-graduate medical training. We report our experience with a required one-week ID consult rotation for Internal Medicine (IM) interns with a focus on antimicrobial stewardship education.

**Methods:**

Since 2018 all IM interns at our institution have participated in a required one-week ID consult rotation. Antimicrobial stewardship is a core feature of this rotation, with educational resources on antibiotic spectrum and decision-making, and interdisciplinary rounding with ID pharmacists. Between March 2020 and May 2021 we piloted an 11-item pre-rotation and post-rotation quiz with distinct but paired questions on key stewardship topics. The quiz was administered anonymously in SurveyMonkey. Mean pre/post rotation scores were compared using a paired T-test and the McNemar test of paired proportions was used to compare the pre/post change in percentage of correct responses for each topic.

**Results:**

Among 47 interns who completed the rotation, 16 interns completed both pre- and post-rotation quizzes (response rate=34%). Mean scores on the pre-rotation quiz were 60%, compared to 77% on the post-rotation quiz (p=0.01), indicating significant improvement at the end of the rotation (Figure 1). Among 11 residents who scored below 65% on their pre-rotation quiz, all achieved an increased score on their post-rotation quiz (mean pre-test of 49% to mean post-test 79%). Table 1 displays the question topics and pre/post test change in percentage correct. The most difficult pre-test topics were ‘Recognition of AmpC-Expressing Organisms’ and ‘Antibiotics with activity against *Pseudomonas aeruginosa*,’ which improved, from 31% to 81% correct (p=0.03) and 50% to 100% correct (p=0.01), respectively.

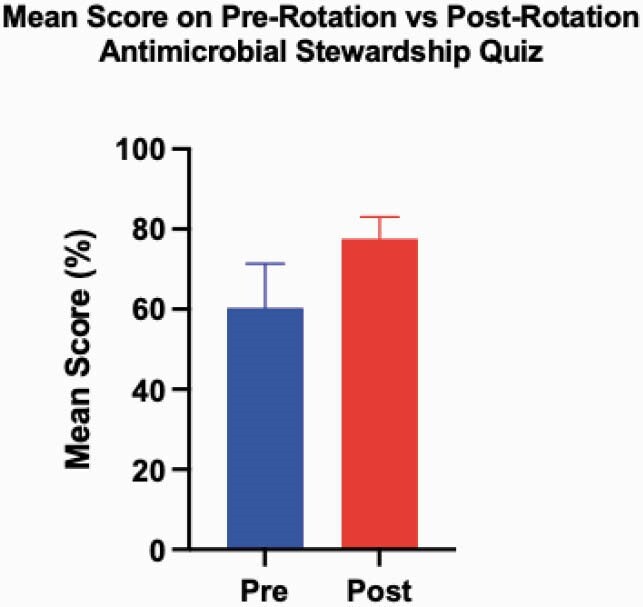

Figure 1. Mean score of interns on pre-rotation vs post-rotation antimicrobial stewardship quiz from March 2020 to May 2021 (n=16; p=0.01).

Table 1. Question topics and change in percentage correct on pre-rotation and post-rotation quizzes.

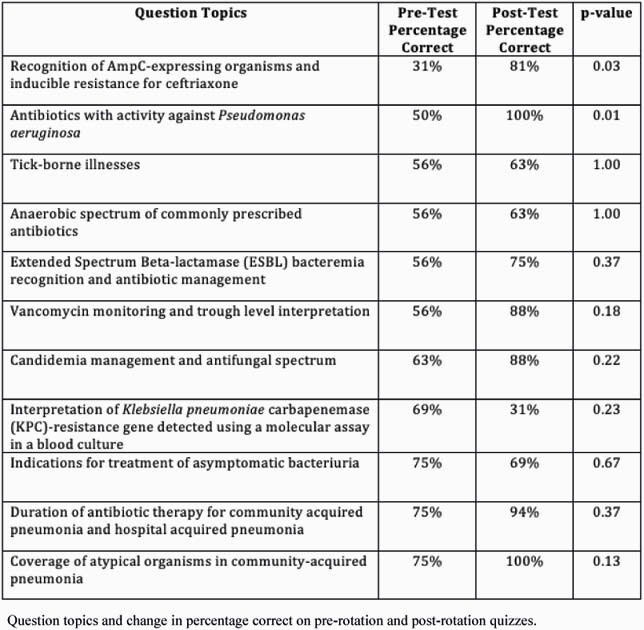

**Conclusion:**

A required one-week ID consult rotation for IM interns improved antimicrobial stewardship knowledge. Our experience may serve as a model for other institutions interested in increasing IM housestaff exposure to ID and antimicrobial stewardship.

**Disclosures:**

**Kristen Marks, MD**, **Gilead Sciences** (Grant/Research Support)

